# Safety assessment of anti-B cell maturation antigen chimeric antigen receptor T cell therapy: a real-world study based on the FDA adverse event reporting system database

**DOI:** 10.3389/fimmu.2024.1433075

**Published:** 2024-09-03

**Authors:** Wei Liu, Shuzhi Lin, Xiaoying Zhu, Lin Yin, Qian Liu, Shuang Lei, Bianling Feng

**Affiliations:** ^1^ The Department of Pharmacy Administration, School of Pharmacy, Xi’an Jiaotong University, Xi’an, Shaanxi, China; ^2^ The Center for Drug Safety and Policy Research, Xi’ an Jiaotong University, Xi’ an, Shaanxi, China

**Keywords:** chimeric antigen receptor T cell therapy, B cell maturation antigen, FDA adverse event reporting system, adverse events, disproportionality analysis

## Abstract

**Background:**

On April 18, 2024, the U.S. Food and Drug Administration officially required updating of the “boxed warning” for T cell malignancies for all chimeric antigen receptor T cell (CAR-T) therapies. Given the clinical significance of these therapies, a rigorous safety assessment is paramount. However, comprehensive real-world safety studies have been lacking for the newly marketed CAR-T products idecabtagene vicleucel (ide-cel) and ciltacabtagene autoleucel (cilta-cel), which target B cell maturation antigen, especially regarding the risk of secondary malignancies. Therefore, we aimed to thoroughly analyze the adverse events (AEs) information in the FDA Adverse Event Reporting System (FAERS) database to comprehensively understand the safety risks of ide-cel and cilta-cel.

**Methods:**

We extracted AE reports related to ide-cel and cilta-cel from the FAERS database (https://fis.fda.gov/extensions/FPD-QDE-FAERS/FPD-QDE-FAERS.html.) from January 1, 2019 to December 31, 2023. Disproportionality analysis and Bayesian analysis were used to identify risk signals across subgroups and specific cases (including for death and secondary malignancies). Weibull distribution analysis was employed to determine the time to AE onset.

**Results:**

A total of 695 AE reports for ide-cel and 848 for cilta-cel were included in the FAERS database. This analysis identified 81 positive signals for ide-cel and 74 for cilta-cel. Notably, comparisons with the drug labels revealed “unexpected signals,” including febrile bone marrow aplasia (reporting odds ratio=69.10; confidence interval 39.12–122.03) and plasma cell myeloma (12.45; 8.18–18.95) for ide-cel, and increased serum ferritin (24.98; 8.0–77.58) and large intestine perforation (18.57; 5.98–57.69) for cilta-cel. Both drugs showed a higher AE incidence among male recipients and patients aged ≥65 years, although female recipients faced a greater risk. Most AEs occurred at the early stage of administration. However, secondary malignancies were detected for both drugs, primarily occurring one-year post-administration.

**Conclusion:**

This study provides a foundation for understanding the safety profile of CAR-T cell therapy, particularly in relation to the emergence of secondary malignancies. Such insights are helpful for clinical decision-making and the safe and effective utilization of these therapeutic agents.

## 1 Introduction

As a novel treatment modality, chimeric antigen receptor T (CAR-T) cell therapies are a breakthrough therapy for a variety of hematological malignancies (1). CAR-T cell therapy targeting B cell maturation antigen (BCMA) relies on the specific recognition of BCMA by a chimeric antigen receptor (CAR) that is introduced into T cells via genetic engineering technology; this therapy makes full use of the immune activity of T cells to accurately attack tumor cells expressing BCMA (2, 3).

The US Food and Drug Administration (FDA) approved idedecabtagene vicleucel (ide-cel) in March 2021, relying on a Phase II (KarMMa) trial for adult patients with relapsed or refractory multiple myeloma (RRMM) (4). This trial demonstrated a 72% overall response rate (ORR), with 28% achieving a stringent complete response (CR) and a median duration of response (DoR) of 11 months (4). Subsequently, in February 2022, the FDA approved ciltacabtagene autoleucel (cilta-cel) based on the Phase II (CARTITUDE-1) trial for adult patients with RRMM, showcasing an ORR of 97.9%, a stringent CR rate of 78.4%, and a median DoR of 21.8 months (5, 6). Both therapies incorporate the same second-generation design of the domain (CD3ζand 4-1BB) targeting BCMA on target cells (7). However, their ectodomains differ, with ide-cel containing a mouse-derived binding domain specific to one BCMA epitope, while cilta-cel with two camelid VH binding domains (8, 9). Studies have indicated that ide-cel and cilta-cel exhibit favorable risk–benefit ratios during treatment, outperforming standard therapy with hazard ratios of 0.49 and 0.26, respectively (10, 11). thereby markedly improving the treatment and prognosis of patients with RRMM (10, 12).

Although CAR-T cell therapies have achieved notable therapeutic efficacy in the realm of hematological oncology, the FDA has recently required updating of the “boxed warning” for T cell malignancies for all CAR-T cell therapies, which are rare but extremely serious adverse reactions (13). In addition, similar to other innovative therapies, CAR-T cell therapy is prone to inducing immune-related serious adverse events (AEs), such as cytokine release syndrome (CRS) and immune effector cell-associated neurotoxicity syndrome (ICANS) (14). Recent studies indicate that the prevalence of AEs is escalating in tandem with increasing clinical utilization, and, alarmingly, the incidence of secondary malignancies stands at a substantial 6% (15). However, for the novel anti-BCMA CAR-T products, ide-cel and cilta-cel, the current research landscape is limited to case reports and lacks a comprehensive safety analysis, especially for secondary malignancies (16, 17). At present, the safety data for the two drugs—primarily from clinical trials—are constrained by limited durations, stringent selection criteria, and a single experimental design, impeding the timely identification of rare AEs (18). Given the crucial role of anti-BCMA CAR-T therapy for the treatment of hematological malignancies, there is still a lack of comprehensive pharmacovigilance studies with large sample sizes or utilizing extensive databases, particularly for AEs not addressed in clinical trials or product inserts.

The FDA Adverse Event Reporting System (FAERS), a database designed to support the FDA’s post-market surveillance of drugs and therapeutic biologic products, gathers a large amount of AE information from actual clinical application, and is currently the largest pharmacovigilance database (19, 20). Disproportionality analysis can identify potential risk signals from the huge amount of AE information in the FAERS database (21). The aim of this study was to comprehensively evaluate the real-world safety profile of anti-BCMA CAR-T cell therapy by analyzing the AEs recorded in the FAERS database, particularly focusing on secondary malignancies. We also sought to examine the uniformity and differences between anti-BCMA CAR-T product-related AEs to provide a more reliable and comprehensive basis for clinical decision-making and to promote the safe, effective use of the drugs.

## 2 Methods

### 2.1 Data source

This study comprises a retrospective pharmacovigilance analysis utilizing the FAERS database to identify potential associations between ide-cel or cilta-cel and AEs. FAERS(https://fis.fda.gov/extensions/FPD-QDE-FAERS/FPD-QDE-FAERS.html.), a vast spontaneous reporting system, encompasses diverse AE reports from healthcare professionals, consumers, and clinical studies. It comprises seven data tables, which include patient demographics, drug information, and AE details (22). Adhering to the International Safety Reporting Guidelines (ICH E2B), FAERS utilizes the International Dictionary of Medical Terms (MedDRA) to standardize AE terminology, ensuring data accuracy (23). This study extracted AE reports involving ide-cel and cilta-cel as “primary suspects” (PS) or “secondary suspects” (SS) from the FAERS database from January 1, 2019 to December 31, 2023. AEs were described and classified according to the preferred term (PT) and system organ class (SOC) in MedDRA terms.

To ensure the accuracy, consistency, and reliability of the data, we cleaned and deduplicated the adverse event data according to the method recommended by the FDA. In accordance with the FDA’s guidance, we identified the most recent FDA_DT as the temporal marker for instances where the PRIMARY_ID was identical. In scenarios where both FDA_DT and CASE_ID were identical, we prioritized selecting the record with the higher PRIMARY_ID in order to discard redundant reports originating from various sources (24, 25).

### 2.2 Statistical analysis

To detect suspicious signals, we used disproportionality analyses, which compare the incidence of an AE among persons who were taking a target drug with those who were not. If AE frequency in the drug population significantly exceeded that in the non-drug population, surpassing a set threshold, a potential association was considered (26). As there is no definitive gold standard for risk signal detection, each method has its merits and limitations (27). The reporting odds ratio (ROR) may show better early signal detection, but its monitoring ability decreases with an increase in reporting time (28). Conversely, the Bayesian confidence propagation neural network, represented by the information component (IC), has a strong ability to detect unique signals even when the number of AEs related to the target drug is small (29). In order to improve the reliability of the results and avoid the interference of false positive signals, we selected ROR and IC analyses to mine suspicious signals. The formulas are provided in **Supplementary Table 1**.

We used descriptive statistics to analyze demographic information related to ide-cel and cilta-cel. To enhance the reliability of the findings, we conducted separate disproportionality analyses based on AE signals. Then, we performed in-depth subgroup analyses, stratifying patients by sex (male and female) and age groups (18-64 years and ≥65 years, choosing 65 as the boundary. Patients aged below18 years were exclude because of insufficient reports) (30). Signals not listed in the drug instructions were labeled “unexpected signals.” Additionally, we extracted AE reports with death outcomes to assess the safety of BCMA products.

### 2.3 Time-to-onset analysis

To understand the timings of ide-cel– and cilta-cel– induced AEs and identify the AEs requiring long-term monitoring, we statistically analyzed the time to onset (TTO) for ide-cel and cilta-cel. In addition, Weibull distribution analysis has the best test performance for assessing AE occurrence period after treatment (31). Therefore, we applied the Weibull distribution to model the TTO of AEs, deriving the shape parameter (β). If β<1, the AE is mainly concentrated in the early stage of administration of the medication; if β=1, the AE occurs randomly with no obvious time aggregation; and β>1 is thought to indicate an increase with time (32). Enhanced monitoring during periods of increased AE incidence may reduce the risk to patients.

## 3 Results

### 3.1 Descriptive analysis

This study extracted 11,900,484 reports from the FAERS database. After data preprocessing, 695 AE reports related to ide-cel and 848 AE reports related to cilta-cel were screened. **Table 1** summarizes the demographic characteristics of these AE reports. Among ide-cel AE reports, we found that over half of patients were male (52.4%), patients aged ≥65 years accounted for the largest proportion (46.6%), and most patients reported weights ranging from 50 to 100 kg (56.5%). Furthermore, the outcome of death accounted for 10.5% of all reports. For cilta-cel, the proportion of male patients was higher than female (35.4% versus 23.9%), patients aged ≥65 years accounted for the largest proportion (20.6%), and the outcome of death accounted for 9.4%. Notably, there were many missing values for “Characteristics” for reports related to cilta-cel, possibly owing to over one-third of the reports coming from consumers (38.9%). In terms of report sources, whether for ide-cel (71.3%) or cilta-cel (55.8%), most reports were provided by healthcare professionals. Additionally, the majority of reports originated from the United States, accounting for 78.4% of ide-cel reports and 84.7% of cilta-cel reports.

**Table 1:** Characteristics of reports with ide-cel and cilta-cel from the FAERS database.

Characteristics	Reports, N (%)	
	Ide-cel	Cilta-cel
Overall	N=676	N=848
Sex		
Female	252 (37.3)	203 (23.9)
Male	354 (52.4)	300 (35.4)
Unknown or missing	70 (10.4)	345 (40.7)
Weight, kg		
<50	10 (1.5)	5 (0.6)
50-100	382 (56.5)	230(27.1)
>100	50 (7.4)	23 (2.7)
Unknown or missing	234 (34.6)	590 (69.6)
Age group, years		
<18	0	2 (0.2)
18-64	232 (34.3)	127 (15.0)
≥65	316 (46.8)	175 (20.6)
Unknown or missing	128 (18.9)	544 (64.2)
Reporter		
Healthcare professionals	482 (71.3)	473 (55.8)
Consumers	84 (12.4)	330 (38.9)
Unknown or missing	110 (16.3)	45 (5.3)
Outcome		
Death	71(10.5)	80(9.4)
Life-threatening	41(6.1)	15(1.8)
Hospitalization (initial or prolonged)	189(28.0)	230(27.1)
Disability	1(0.1)	1(0.1)
Other	251(37.1)	162(19.1)
Characteristics	Reports, N (%)	Characteristics
	Ide-cel	Cilta-cel
Unknown or missing	123(18.2)	360(42.5)
Report year		
2019	4(0.6)	0(0)
2021	113(16.7)	13(1.5)
2022	285(42.2)	184(21.7)
2023	274(40.5)	651(76.8)
Reporting country		
United States	530(78.4)	718 (84.7)
Other country	145(21.5)	130(15.3)
Unknown or missing	1(0.1)	0

Ide-cel, Idecabtagene vicleucel; Cilta-cel, Ciltacabtagene autoleucel.

### 3.2 Disproportionality signals at the SOC level

Utilizing the criteria of ROR or IC, we identified significant associations of SOCs affected by AEs linked with ide-cel and cilta-cel. Statistical analysis revealed that 23 SOCs were impacted by AEs related to ide-cel, with significant SOCs for ide-cel including immune system disorders, blood and lymphatic system disorders, nervous system disorders, investigations, metabolism and nutrition disorders, and vascular disorders. Notably, immune system disorders and blood and lymphatic system disorders met both ROR and IC criteria (**Supplementary Table 2**). For cilta-cel, 23 SOCs were involved, with immune system disorders being the only SOC that satisfied both ROR and IC standards. Importantly, signals meeting only the ROR criteria, such as nervous system disorders, infections and infestations, blood and lymphatic system disorders, surgical and medical procedures, and product issues, may also indicate important and frequently occurring AEs (**Supplementary Table 3**). These findings highlight the most common SOCs associated with AEs induced by ide-cel and cilta-cel, thereby pinpointing areas that merit further clinical scrutiny and research.

### 3.3 Disproportionality signals at the PT level and for subgroups

We identified 81 positive signals associated with ide-cel and 74 signals with cilta-cel. **Tables 2**, **3** present the top 20 signals ranked by ROR values for ide-cel and cilta-cel, and indicate the strongest associations with the target drugs. The most common AEs associated with ide-cel include CRS (n=411), fatigue (n=151), and ICANS (n=100). The signal with the highest ROR value was alanine aminotransferase (ROR=1086.3; confidence interval [CI] 527.35–2237.73). Notably, comparison with the drug instructions (33) revealed “unexpected signals” for ide-cel, such as febrile bone marrow aplasia (ROR=69.1; CI 39.12–122.03), myelodysplastic syndrome (ROR=12.93; CI 5.80–28.83), and hepatotoxicity (ROR=13.86; CI 5.76–33.34) (complete information in **Supplementary Table 4**).

**Table 2:** Signal strength of ide-cel associated reports at the preferred terms level (top 20).

Preferred term (PT)	Cases (n)	ROR (95%CI)	IC (IC025)
Alanine aminotransferase	8	1086.3(527.35-2237.73)	9.96(8.27)
Lymphocyte adoptive therapy	11	871.65(472.71-1607.28)	9.67(7.99)
Cytokine release syndrome	411	691.40(620.44-770.48)	9.10(7.43)
Immune effector cell-associated neurotoxicity syndrome	100	568.28(463.27-697.09)	9.03(7.36)
Alanine aminotransferase abnormal	22	268.82(175.90-410.82)	8.03(6.36)
Hypogammaglobulinaemia	32	133.18(93.80-189.11)	7.02(5.36)
Pseudomonal bacteraemia	3	86.78(27.86-270.29)	6.43(4.76)
Hypoalbuminaemia	22	86.50(56.77-131.82)	6.41(4.74)
Neurotoxicity	55	75.10(57.43-98.19)	6.19(4.52)
Febrile bone marrow aplasia	12	69.10(39.12-122.03)	6.10(4.43)
Haemophagocytic lymphohistiocytosis	16	39.72(24.28-64.99)	5.30(3.63)
Plasma cell myeloma recurrent	6	39.62(17.76-88.38)	5.30(3.63)
Cytopenia	21	37.11(24.13-57.06)	5.20(3.53)
Haematotoxicity	13	36.87(21.36-63.65)	5.19(3.53)
Neutrophil count abnormal	4	34.64(12.97-92.50)	5.11(3.44)
Immunodeficiency	22	33.64(22.09-51.21)	5.06(3.39)
Aspartate aminotransferase increased	47	31.89(23.88-42.58)	4.96(3.30)
Laryngeal oedema	5	26.57(11.04-63.96)	4.73(3.06)
Torsade de pointes	6	24.47(10.97-54.57)	4.61(2.94)
Altered state of consciousness	19	24.26(15.44-38.12)	4.59(2.92)

ROR, reporting odds ratio; 95%CI, 95% confidence interval; IC, information component; IC025: 95% confidence interval of IC.

**Table 3:** Signal strength of cilta-cel associated reports at the preferred terms level (top 20).

Preferred term (PT)	Cases (n)	ROR (95%CI)	IC (IC025)
Haemophilus sepsis	3	2877.52(854.21-9693.36)	11.29(9.52)
Iiird nerve disorder	4	1448.72(523.71-4007.56)	10.39(8.68)
Lymphocyte adoptive therapy	11	1301.15(705.17-2400.84)	10.24(8.56)
Metapneumovirus pneumonia	3	1251.10(388.70-4026.84)	10.20(8.48)
Cranial nerve paralysis	5	950.87(386.86-2337.19)	9.82(8.13)
Facial nerve disorder	7	650.35(305.72-1383.48)	9.29(7.61)
Bell’s palsy	18	397.03(248.32-634.80)	8.59(6.92)
Immune effector cell-associated neurotoxicity syndrome	45	366.85(272.02-494.74)	8.45(6.78)
Cytokine release syndrome	158	353.52(299.59-417.17)	8.29(6.62)
Parkinsonism	22	104.22(68.35-158.92)	6.68(5.01)
Physical product label issue	3	100.26(32.21-312.12)	6.64(4.97)
Alanine aminotransferase abnormal	5	89.00(36.92-214.57)	6.46(4.79)
Peripheral motor neuropathy	3	87.46(28.10-272.17)	6.44(4.77)
Chronic inflammatory demyelinating polyradiculoneuropathy	3	78.51(25.24-244.26)	6.29(4.62)
Haematological infection	3	76.12(24.47-236.81)	6.24(4.57)
Facial paresis	6	75.37(33.76-168.28)	6.22(4.56)
Neurotoxicity	34	68.86(48.99-96.78)	6.07(4.40)
Facial paralysis	21	67.92(44.13-104.55)	6.06(4.39)
Haemophagocytic lymphohistiocytosis	18	66.89(42.00-106.53)	6.04(4.37)
Product label issue	11	58.69(32.41-106.30)	5.86(4.19)

ROR, reporting odds ratio; 95%CI, 95% confidence interval; IC, information component; IC025: 95% confidence interval of IC.

For cilta-cel, frequently occurring AEs include CRS (n=158), pyrexia (n=58), and ICANS (n=45). Additionally, strong associations were observed with *Haemophilus* sepsis (ROR=2877.52; CI 854.21–9693.36). We also detected AE signals not mentioned in the drug instructions (34), including physical product label issue (ROR=100.26; CI 32.21–312.12), large intestine perforation (ROR=18.57; CI 5.98–57.69), and blood lactate dehydrogenase increased (ROR=16.27; CI 6.76–39.16) (complete information in **Supplementary Table 5**).

To mitigate the potential confounding of signal mining outcomes by demographic factors, we conducted a disproportionality analysis for various subgroups and calculated the ROR of AE exposure based on sex and age groups (18-64 and ≥65 years). For ide-cel, CRS, fatigue, and ICANS were the most frequently reported in all age groups. Among the top 15 AEs reported across age groups, acute kidney injury, parkinsonism, and sepsis were more prevalent in patients aged 18-64 years whereas alanine aminotransferase abnormal, hemophagocytic lymphohistiocytosis, and headache were more common in older patients. Notably, signals for parkinsonism and transaminases increased were detected exclusively in male patients aged 18-64 years, whereas torsade de pointes was observed only in female patients aged ≥65 years. (**Supplementary Figures 1**, **2**).

A similar subgroup analysis was conducted for cilta-cel. CRS, pyrexia, and ICANS were the most reported AEs across age groups. Pneumonia, febrile neutropenia, and acute kidney injury were more prevalent in patients aged 18-64 years, while parkinsonism, septic shock, and third nerve disorder were more common in patients aged ≥65 years. Additionally, Guillain-Barre syndrome and parkinsonism were detected as signals only in male patients (**Supplementary Figures 3, 4**).

### 3.4 In-depth analysis of secondary malignancies

Furthermore, given the severity and rarity of secondary malignancies associated with CAR-T cell therapy during clinical trials and practical application, we conducted a thorough analysis. Owing to the limited number of reports on secondary malignancy AEs, we employed IC analysis, which is effective for detecting important signals even with limited AE data, to monitor risk signals for secondary malignancies.

**Figure 1:**
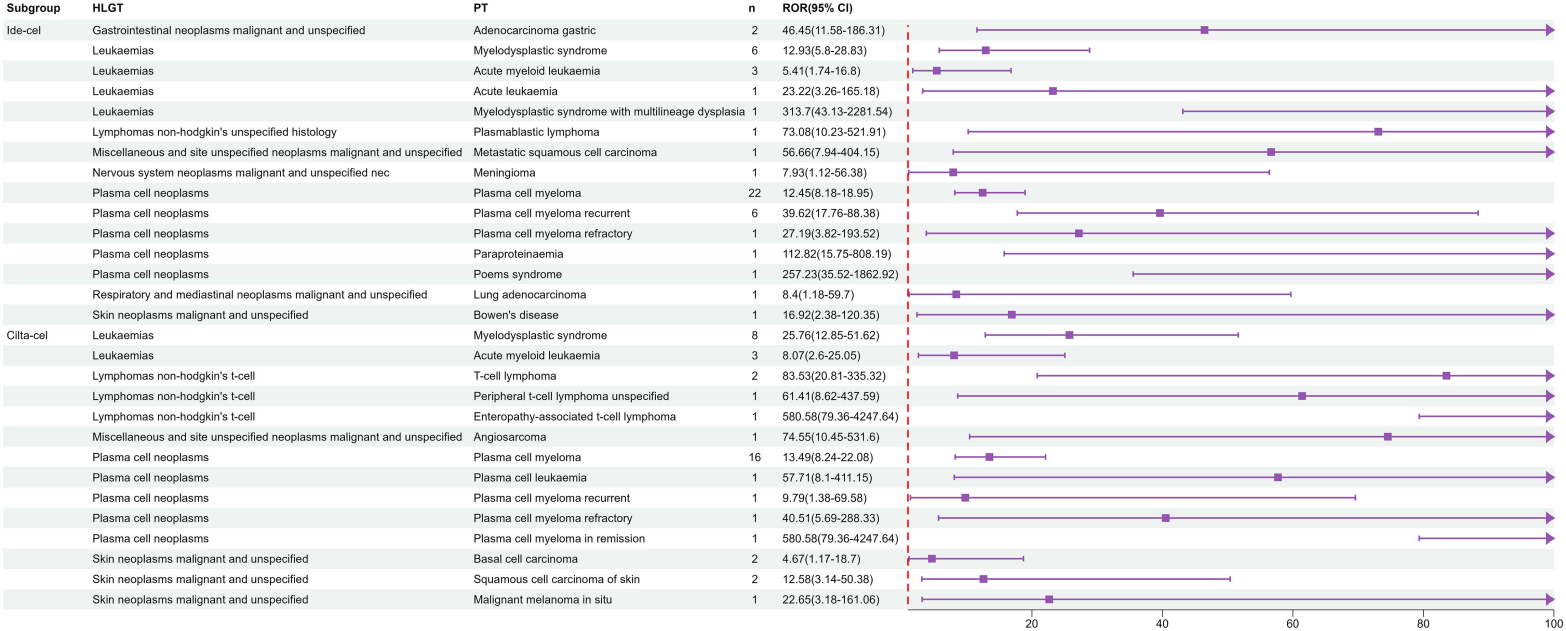
Risk signals of secondary malignancies with ide-cel and cilta-cel.

At the high-level group term level, significant signals for ide-cel included gastrointestinal neoplasms malignant and unspecified, leukemias, and lymphomas non-hodgkin’s unspecified histology. For cilta-cel, significant AE signals included those for leukemias, lymphomas non-hodgkin’s t-cell, and plasma cell neoplasms. As shown in **Figure 1**, leukemias, plasma cell neoplasms, skin neoplasms malignant and unspecified, and miscellaneous and site unspecified neoplasms malignant and unspecified were common signals for both drugs. Notably, we only detected signals for T cell malignancies for cilta-cel.

**Figure 2:**
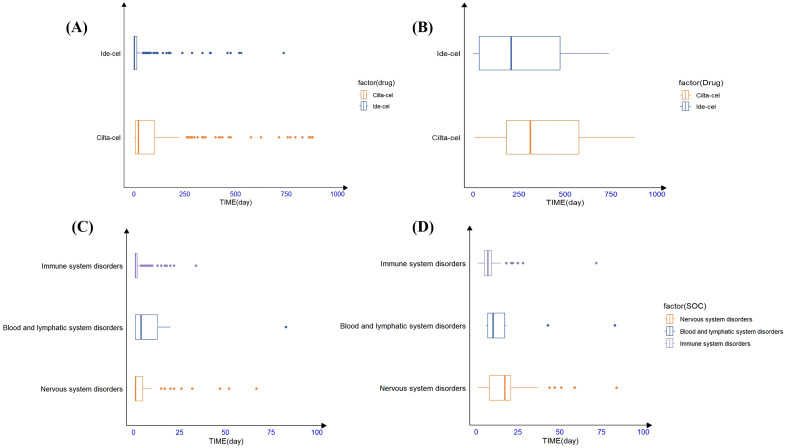
The timing of AE occurrence for ide-cel and cilta-cel; **(A)** The time to onset of all of AEs for ide-cel and cilta-cel. **(B)** The time to onset of secondary malignancies for ide-cel and cilta-cel. **(C)** The time to onset of AEs occurred at the SOC level for ide-cel. **(D)** The time to onset of AEs occurred at the SOC level for cilta-cel.

Additionally, TTO analysis of secondary malignancies revealed that the mean time to malignancy onset for ide-cel was 498.9 days with a median of 205.5 days, whereas for cilta-cel, the mean time was 390 days with a median of 311 days (**Figure 2B**). The identified malignancy risk signals and their temporal patterns provide deeper insights into the clinical safety profiles of these two drugs.

### 3.5 Gender difference risk signals

**Figure 3:**
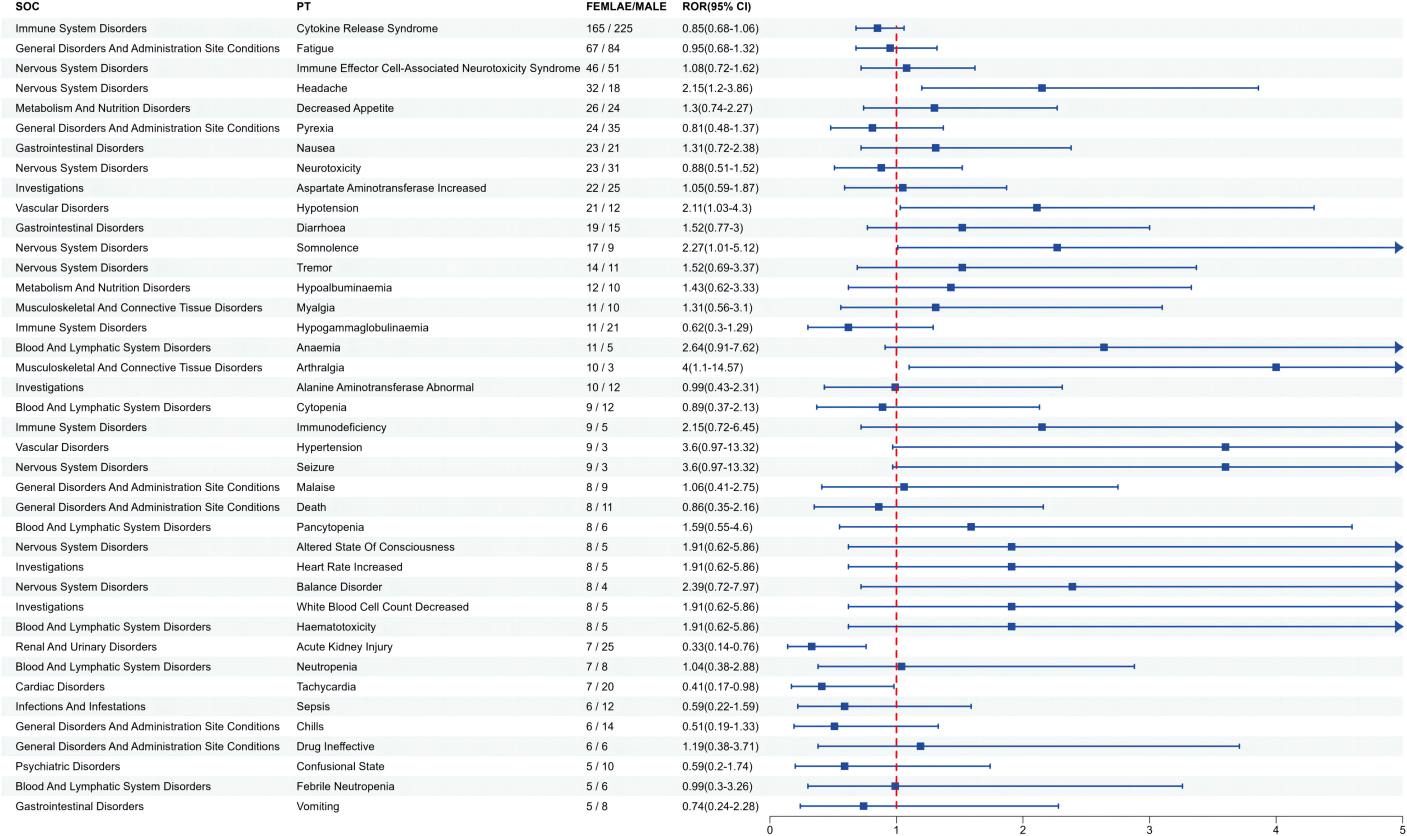
Gender difference risk signals of ide-cel.

**Figure 4:**
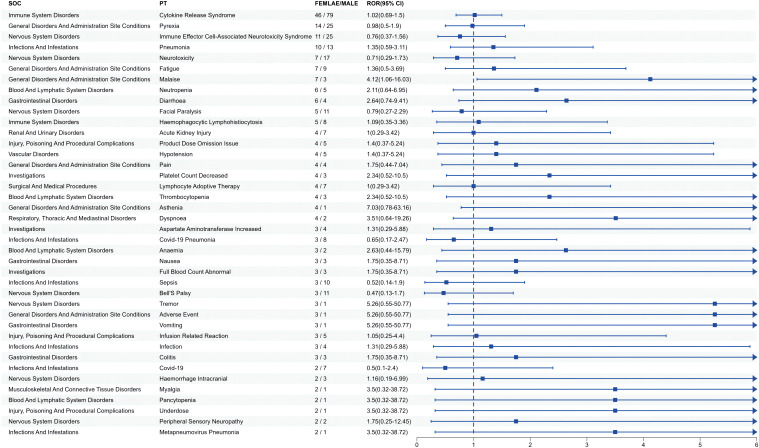
Gender difference risk signals of cilta-cel.

Since gender differences were present at the overall AE reporting level, we further analyzed sex differences in the mined AE results. **Figure 3** shows that for ide-cel, female patients were more likely than male patients to experience most of the AEs. The most likely AEs in women included arthralgia, hypertension, seizure, anemia, and balance disorder. Men were most likely to develop sepsis, acute kidney injury, chills, tachycardia and hypogammaglobulinemia. Similarly, **Figure 4** shows that, among all the AEs for cilta-cel, women were more prone than men to experience most kinds of AEs, including asthenia, tremor, vomiting, and dyspnea. Men were most likely to suffer from confusion, coronavirus disease 2019 (COVID-19), sepsis, Bell’s palsy, and neurotoxicity.

### 3.6 Death outcome in-depth analysis

In AE reports with the outcomes of death for ide-cel, male patients accounted for a higher proportion (52.1%) than female patients (36.6%), and there was a slightly higher proportion of patients aged ≥65 years than those aged 18–64 years. For cilta-cel, male patients also comprised a larger share (50.0%) than female patients (16.3%), with over one-third aged ≥65 years (33.8%). Most of the reports for both drugs originated from the United States (63.4% and 81.3%) (**Supplementary Table 6**).

**Table 4** shows the top 10 AEs with the highest number of correlations with ide-cel and cilta-cel death outcomes at the PT level. The statistical results showed that CRS and ICANS were the most common AEs associated with death outcomes for ide-cel and cilta-cel, and had the strongest correlations with death outcomes. We also found that other AEs with strong correlations with ide-cel–related death were hemophagocytic lymphohistiocytosis, hypogammaglobulinemia, and parkinsonism. Hemophagocytic lymphohistiocytosis, COVID-19 pneumonia, and neurotoxicity were also strongly correlated with cilta-cel–related death.

**Table 4:** Death outcome-related AEs of Ide-cel and Cilta-cel (Top 10).

Preferred term (PT)	Cases(n)	ROR (95%CI)	IC (IC025)
Ide-cel			
Cytokine release syndrome	27	677.61(453.57-1012.31)	9.22(7.55)
Immune effector cell-associated neurotoxicity syndrome	17	1663.50(1013.87-2729.37)	10.58(8.90)
Sepsis	15	37.16(22.03-62.70)	5.12(3.45)
Haemophagocytic lymphohistiocytosis	8	246.57(121.78-499.24)	7.89(6.22)
Acute kidney injury	6	8.14(3.62-18.31)	2.99(1.32)
Plasma cell myeloma	6	36.07(16.04-81.14)	5.14(3.46)
Preferred term (PT)	Cases(n)	ROR (95%CI)	IC (IC025)
Neurotoxicity	5	83.69(34.50-203.01)	6.36(4.68)
Parkinsonism	5	144.40(59.52-350.34)	7.14(5.47)
Hypogammaglobulinaemia	4	206.85(76.95-556.07)	7.67(5.99)
Cilta-cel			
Cytokine release syndrome	21	585.87(373.00-920.21)	9.04(7.36)
Immune effector cell-associated neurotoxicity syndrome	14	1537.53(892.55-2648.59)	10.48(8.80)
Covid-19 pneumonia	7	184.51(86.82-392.12)	7.48(5.80)
Sepsis	7	18.98(8.93-40.33)	4.20(2.52)
Haemophagocytic lymphohistiocytosis	6	207.76(92.21-468.09)	7.66(5.98)
Pneumonia	6	5.34(2.37-12.03)	2.38(0.71)
Pyrexia	6	5.18(2.30-11.66)	2.34(0.66)
Respiratory failure	6	20.41(8.40-49.57)	4.32(2.64)
Neurotoxicity	5	75.48(28.05-203.06)	6.21(4.53)
Encephalopathy	4	50.01(18.59-134.54)	5.62(3.94)

ROR, reporting odds ratio; 95%CI, 95% confidence interval; IC, information component; IC025: 95% confidence interval of IC.

### 3.7 AE TTO

Statistical analysis revealed that the majority of AEs associated with ide-cel (83.2%) and cilta-cel (56.25%) occurred within the first 30 days following treatment. For ide-cel, the median onset time for AEs was 2 days, with a noteworthy proportion (47.7%) occurring on the day of administration. For cilta-cel, the median onset time for AEs was 21.50 days. To further understand the timing of AE occurrence at the SOC level for both drugs, we conducted an analysis based on common positive SOC signals. **Figure 2** visually presents the statistical results in the form of box plots, providing insights into the distribution of the data and outliers. The results indicated that, at the SOC level, the onset of corresponding SOC AEs occurred within 30 days of treatment (**Figure 2A**). Additionally, the Weibull distribution analysis revealed that β<1 for both drugs, indicating an early failure pattern, in which the majority of AEs occurred during the initial stages of treatment. (**Table 5**).

**Table 5:** Weibull distribution of ide-cel and cilta-cel.

Database	Case (n)	Scale parameter α (95% CI)	Shape parameter β (95% CI)	Type
Ide-cel	676	12.67(9.11,16.22)	0.43(0.40,0.46)	Early failure type
Cilta-cel	848	74.08(56.58,91.58)	0.61(0.55,0.67)	Early failure type

When the shape parameter β<1 is 95%CI<1, the incidence of AE is considered to decline over time (early failure type).

Notably, we found many AEs that emerged one year after drug treatment (**Supplementary Table 7**). Among those associated with ide-cel were myelodysplastic syndrome, Bowen’s disease, bladder cancer, metastatic squamous cell carcinoma, and squamous cell carcinoma of the skin. For cilta-cel, AEs that occurred one year after treatment included myelodysplastic syndrome, basal cell carcinoma, prostate cancer, malignant melanoma, and transient ischemic attack.

## 4 Discussion

We conducted a retrospective pharmacovigilance study utilizing the FAERS database to evaluate BCMA-targeted CAR-T cell therapies, focusing on novel potential safety signals, the safety profiles for secondary malignancy, subgroup signal variations, and the time distribution of AE occurrence. These findings are intended to inform and shape clinical safety practices.

Our study found that CRS and ICANS were the most frequently occurring AEs associated with ide-cel and cilta-cel (**Figures 2C, D**), exhibiting significant signals and the strongest correlations with death. These findings were consistent with the drug instructions and clinical trial results, thereby validating the reliability of our research outcomes (11, 15, 35–37). At the SOC level, the incidence of Nervous system disorders associated with cilta-cel (n=316, 46.74%) and ide-cel (n=381, 44.9%) was substantial, each approximating half of their respective AE reports. This highlights a critical clinical concern and emphasizes the vital need for rigorous monitoring of neurological symptoms in patients treated with either drug.

Notably, performing a disproportionality analysis and comparing the results with the drug instructions, we uncovered “unexpected signals” for ide-cel, such as plasma cell myeloma and hepatotoxicity, and for cilta-cel, including large intestine perforation and blood lactate dehydrogenase increased. These findings indicate new potential safety risks associated with these drugs in real-world settings. Additionally, we discovered that the only AEs significantly associated with cilta-cel but not with ide-cel were Guillain-Barre syndrome and COVID-19 pneumonia. It has been reported that patients receiving cilta-cel treatment may have an increased risk of COVID-19 pneumonia (11). Furthermore, the median TTO for ide-cel–related AEs was 2 days, whereas that for cilta-cel–related AEs was 21.50 days. This difference may be attributable to the varying dosages used for the two drugs, as cilta-cel is generally administered at a dosage that is 10%–15% of that for ide-cel, potentially resulting in the induction of distinct AEs (37).

In particular, we observed a risk of secondary malignancies associated with both ide-cel and cilta-cel. In particular, cilta-cel has a risk signal for T cell malignancies, a rare yet severe AE associated with CAR-T cell therapy. The FDA has issued a boxed warning for T cell malignancies across CAR-T cell therapies. Although these reports constitute a minority of cases, our signal mining results indicate an increasing risk of malignancy. This concurs with prior studies documenting disproportionately high frequencies of myeloid and T cell tumors associated with CAR-T therapies (38). Based on the TTO analysis of AEs, we found that secondary malignancies typically occur after one year of treatment. Prior studies have shown that anti-BCMA CAR-T products can induce protracted adverse reactions, even leading to relapse in patients who had initially responded well. This has been attributed to factors such as the characteristics of myeloma tumor cells, CAR-T cell properties, and the tumor microenvironment (38). Some studies have proposed that since both ide-cel and cilta-cel target only one antigen, BCMA, CAR-T cells may experience persistent insufficiency, resulting in decreased antigen-targeting ability and subsequent tumor escape (39). Researchers are actively exploring methods to improve the safety of CAR-T products (39, 40). In clinical practice, this finding serves as a reminder of the need for long-term, continuous monitoring for malignancy occurrence.

Furthermore, we observed a higher proportion of male patients receiving both drugs, yet it was female patients who exhibited a greater risk of AEs. Research has shown significant individual differences in the pharmacokinetics of ide-cel and cilta-cel (41). However, analysis of death as an outcome revealed that male patients had a higher mortality rate than female patients. This observation could potentially be attributed to unhealthy habits, such as smoking and alcohol consumption, among male patients, which increase the likelihood of complications and result in a poorer prognosis for male patients receiving CAR-T cell therapy (30). Furthermore, we analyzed AEs at the PT level in each subgroup and identified both similarities and differences in signals across subgroups. As the clinical application of CAR-T cell therapy continues to expand, this information is crucial for more refined management based on specific patient characteristics.

Our analysis revealed that the majority of AEs associated with ide-cel and cilta-cel occurred during the initial phase of treatment, with the highest risk occurring within the first 15 days. The Weibull distribution analysis confirmed an early failure pattern, corroborating our statistical AE TTO findings. Consistent with the findings of Vigibase research, we observed that common AEs like CRS and ICANS typically emerged within the first week of CAR-T cell administration (42). Additionally, both drugs had outlier values, indicating delayed AEs. In particular, secondary malignancies emerged, on average, more than a year after treatment. Hence, close monitoring for AEs during the early stages of CAR-T therapies and long-term surveillance for delayed AEs are crucial in clinical practice.

This study has limitations. Firstly, the FAERS database is a self-reporting system, with the inherent limitation of the underreporting of AEs (25). Secondly, while FAERS reports are informative, the reporting rate is influenced by various factors, including the severity of an AE and public perception, rendering the drug–AE causal relationship uncertain (43–45). To enhance reliability, we integrated clinical trial and analogous study results for comparative analysis. Thirdly, the disproportionality analysis can only provide preliminary evidence indicating that there may be an association between drugs and AEs, but it may be affected by confounding factors and it cannot determine whether the association is causal (27). Therefore, when applying the method, we performed subgroup analysis at the level of demographic characteristics to ensure the accuracy of the results. Although not definitive, this study’s comprehensive safety analysis of AE signals associated with BCMA-targeted CAR-T cell therapies could lay the foundation for safe clinical use in the future.

This study validated clinical trial findings on AEs related to ide-cel and cilta-cel administration using the FAERS database, and identified previously unknown or underestimated risks associated with both drugs, thereby complementing existing research. The results of the disproportionality analysis of secondary malignancies indicated that the risk of secondary malignancy is increasing. In addition, the comparison of the signals between the subgroups provides key information for more refined clinical management in the future. In conclusion, this study highlights the importance of long-term ongoing safety studies to identify emerging AEs and potential risk signals associated with the use of ide-cel and cilta-cel, with the potential to promote safe and rational use in clinical settings.

## Data Availability

Publicly available datasets were analyzed in this study. This data can be found here: https://fis.fda.gov/extensions/FPD-QDE-FAERS/FPD-QDE-FAERS.html.
