# Supplementary material for: Safety assessment of anti-B cell maturation antigen chimeric antigen receptor T cell therapy: a real-world study based on the FDA adverse event reporting system database
Source: Front Immunol. 2024 Sep 3;15:1433075. doi: 10.3389/fimmu.2024.1433075 (PMC11405296; doi:10.3389/fimmu.2024.1433075)
Supplement: Supplementary file 1 [file DataSheet1.docx]

# *Supplementary Material*

**Supplementary Table 1** The detailed formulas for disproportionality analysis.

| **Algorithms** | **Equation** | **Threshold** |
| --- | --- | --- |
| ROR | $ROR=\frac{a/c}{b/d}$ | lower limit of 95% CI > l, a≥ 3 |
|  | $95\%CI=e^{ln(ROR)\pm1.96\sqrt{(\frac{1}{a}+\frac{1}{b}+\frac{1}{c}+\frac{1}{d})}}$ |  |
| BCPNN | $IC=={log}_{2}\frac{a(a+b+c+d)}{(a+b)(a+c)}$ | IC025>0 |
|  | ${IC025=log}_{2}\frac{a+0.5}{E+0.5}-{3.3(a+0.5)}^{-1/2}-{2(a+0.5)}^{-3/2}$ |  |

Notes: ROR, reporting odds ratio; 95%CI, 95% confidence interval; IC, information component; BCPNN, bayesian confidence propagation neural network.

Equation: a, the number of target adverse events reports of the target drug; b, The number of other adverse events reports of the target drug; c, the number of target adverse events reports of non-target drugs; d, the number of other adverse events reports of non-target drugs; E:(a+b)(a+c)/(a+b+c+d).

**Supplementary Table 2** Signal strength of ide-cel associated reports at the SOC level

| SOC | Cases(n) | ROR | RORL | IC025 |
| --- | --- | --- | --- | --- |
| Immune system disorders | 486 | 20.91 | 18.93 | 2.40 |
| Nervous system disorders | 381 | 2.32 | 2.08 | -0.59 |
| General disorders and administration site conditions | 349 | 0.81 | 0.72 | -1.92 |
| Investigations | 211 | 1.58 | 1.37 | -1.05 |
| Blood and lymphatic system disorders | 157 | 4.22 | 3.59 | 0.33 |
| Infections and infestations | 116 | 0.89 | 0.74 | -1.82 |
| Gastrointestinal disorders | 115 | 0.58 | 0.48 | -2.41 |
| Metabolism and nutrition disorders | 96 | 2.07 | 1.69 | -0.64 |
| Vascular disorders | 66 | 1.48 | 1.16 | -1.11 |
| Cardiac disorders | 58 | 1.21 | 0.93 | -1.40 |
| Neoplasms benign, malignant and unspecified (incl cysts and polyps) | 56 | 0.71 | 0.54 | -2.15 |
| Musculoskeletal and connective tissue disorders | 54 | 0.43 | 0.33 | -2.83 |
| Respiratory, thoracic and mediastinal disorders | 52 | 0.47 | 0.36 | -2.71 |
| Renal and urinary disorders | 47 | 0.98 | 0.74 | -1.69 |
| Psychiatric disorders | 46 | 0.35 | 0.26 | -3.14 |
| Injury, poisoning and procedural complications | 36 | 0.12 | 0.08 | -4.63 |
| Hepatobiliary disorders | 17 | 0.88 | 0.54 | -1.86 |
| Surgical and medical procedures | 13 | 0.39 | 0.23 | -3.00 |
| Skin and subcutaneous tissue disorders | 4 | 0.03 | 0.01 | -6.77 |
| Social circumstances | 2 | 0.18 | 0.05 | -4.10 |
| Product issues | 1 | 0.02 | 0.00 | -7.04 |
| Eye disorders | 1 | 0.02 | 0.00 | -7.17 |
| Ear and labyrinth disorders | 1 | 0.10 | 0.01 | -5.02 |

Notes: SOC, system organ class; RORL，ROR 95% confidence interval lower limit; IC, information component; IC025: 95% confidence interval of IC.

**Supplementary Table 3** Signal strength of cilta-cel associated reports at the SOC level

| SOC | Cases(n) | ROR | RORL | IC025 |
| --- | --- | --- | --- | --- |
| Nervous system disorders | 316 | 3.01 | 2.66 | -0.29 |
| General disorders and administration site conditions | 279 | 1.00 | 0.87 | -1.67 |
| Infections and infestations | 201 | 2.51 | 2.16 | -0.46 |
| Immune system disorders | 184 | 10.59 | 9.08 | 1.58 |
| Investigations | 107 | 1.17 | 0.96 | -1.46 |
| Gastrointestinal disorders | 61 | 0.45 | 0.35 | -2.75 |
| Blood and lymphatic system disorders | 61 | 2.37 | 1.83 | -0.46 |
| Surgical and medical procedures | 49 | 2.27 | 1.71 | -0.51 |
| Neoplasms benign, malignant and unspecified (incl cysts and polyps) | 47 | 0.89 | 0.67 | -1.83 |
| Injury, poisoning and procedural complications | 43 | 0.21 | 0.15 | -3.80 |
| Respiratory, thoracic and mediastinal disorders | 41 | 0.56 | 0.41 | -2.48 |
| Product issues | 38 | 1.38 | 1.00 | -1.21 |
| Cardiac disorders | 27 | 0.83 | 0.57 | -1.93 |
| Musculoskeletal and connective tissue disorders | 27 | 0.32 | 0.22 | -3.26 |
| Metabolism and nutrition disorders | 21 | 0.66 | 0.43 | -2.26 |
| Vascular disorders | 21 | 0.69 | 0.45 | -2.19 |
| Renal and urinary disorders | 19 | 0.59 | 0.37 | -2.42 |
| Psychiatric disorders | 18 | 0.20 | 0.13 | -3.92 |
| Eye disorders | 14 | 0.45 | 0.27 | -2.79 |
| Skin and subcutaneous tissue disorders | 8 | 0.08 | 0.04 | -5.20 |
| Hepatobiliary disorders | 4 | 0.31 | 0.11 | -3.37 |
| Endocrine disorders | 1 | 0.24 | 0.03 | -3.75 |
| Social circumstances | 1 | 0.14 | 0.02 | -4.53 |

Notes: SOC, system organ class; RORL，ROR 95% confidence interval lower limit; IC, information component; IC025: 95% confidence interval of IC.

**Supplementary Table 4** Signal strength of ide-cel associated reports at the PT level

| SOC | Preferred terms (PT) | Cases  (n) | ROR | 95% CI | |
| --- | --- | --- | --- | --- | --- |
|  |  |  |  | Low bound | Up  bound |
| Blood and lymphatic system disorders | Anaemia | 25 | 3.83 | 2.58 | 5.68 |
|  | Bone marrow failure* | 4 | 4.97 | 1.86 | 13.26 |
|  | Cytopenia | 21 | 37.11 | 24.13 | 57.06 |
|  | Disseminated intravascular coagulation | 7 | 17.94 | 8.54 | 37.69 |
|  | Febrile bone marrow aplasia* | 12 | 69.10 | 39.12 | 122.03 |
|  | Febrile neutropenia | 11 | 4.31 | 2.39 | 7.80 |
|  | Haematotoxicity | 13 | 36.87 | 21.36 | 63.65 |
|  | Myelosuppression | 6 | 5.07 | 2.28 | 11.31 |
|  | Pancytopenia | 17 | 9.07 | 5.62 | 14.61 |
|  | Thrombocytopenia | 13 | 3.23 | 1.87 | 5.58 |
| Cardiac disorders | Arrhythmia | 6 | 3.76 | 1.69 | 8.39 |
|  | Tachycardia | 27 | 8.64 | 5.91 | 12.63 |
|  | Torsade de pointes | 6 | 24.47 | 10.97 | 54.57 |
| General disorders and administration site conditions | Adverse event | 13 | 4.09 | 2.37 | 7.06 |
|  | Chills | 20 | 4.85 | 3.12 | 7.53 |
|  | Fatigue | 151 | 5.10 | 4.32 | 6.01 |
|  | Pyrexia | 61 | 4.98 | 3.86 | 6.43 |
| Hepatobiliary disorders | Hepatotoxicity* | 4 | 13.86 | 5.76 | 33.34 |
|  | Hyperbilirubinaemia | 5 | 4.40 | 1.65 | 11.73 |
| Immune system disorders | Cytokine release syndrome | 411 | 691.40 | 620.44 | 770.48 |
|  | Haemophagocytic lymphohistiocytosis | 16 | 39.72 | 24.28 | 64.99 |
|  | Hypogammaglobulinaemia | 32 | 133.18 | 93.80 | 189.11 |
|  | Immunodeficiency | 22 | 33.64 | 22.09 | 51.21 |
|  | Sarcoidosis* | 3 | 17.54 | 5.65 | 54.48 |
| Infections and infestations | Bacteraemia | 4 | 9.04 | 3.39 | 24.12 |
|  | Clostridium difficile colitis | 4 | 10.60 | 3.97 | 28.28 |
|  | Gastroenteritis* | 4 | 7.93 | 2.97 | 21.15 |
|  | Osteomyelitis | 4 | 5.96 | 2.23 | 15.90 |
|  | Pseudomonal bacteraemia | 3 | 86.78 | 27.86 | 270.29 |
|  | Rhinovirus infection | 4 | 22.86 | 8.57 | 61.02 |
|  | Sepsis | 19 | 4.74 | 3.02 | 7.45 |
|  | Viral upper respiratory tract infection | 4 | 14.05 | 5.27 | 37.50 |
| Investigations | Alanine aminotransferase | 8 | 1086.30 | 527.35 | 2237.73 |
|  | Alanine aminotransferase abnormal | 22 | 268.82 | 175.90 | 410.82 |
|  | Alanine aminotransferase increased | 11 | 5.98 | 3.31 | 10.82 |
|  | Aspartate aminotransferase increased | 47 | 31.89 | 23.88 | 42.58 |
|  | Blood lactate dehydrogenase increased* | 4 | 8.73 | 3.27 | 23.28 |
|  | Blood lactic acid increased | 3 | 15.70 | 5.06 | 48.76 |
|  | Full blood count decreased | 5 | 5.64 | 2.34 | 13.56 |
|  | Heart rate increased | 21 | 6.11 | 3.98 | 9.39 |
|  | Laboratory test abnormal | 6 | 4.42 | 1.99 | 9.86 |
|  | Neutrophil count abnormal | 4 | 34.64 | 12.97 | 92.50 |
|  | Platelet count abnormal | 3 | 11.19 | 3.60 | 34.73 |
|  | Transaminases increased | 4 | 4.67 | 1.75 | 12.47 |
|  | Troponin increased | 4 | 15.10 | 5.66 | 40.30 |
|  | White blood cell count decreased | 21 | 4.81 | 3.13 | 7.39 |
| Injury, poisoning and procedural complications | Infusion related reaction | 11 | 4.11 | 2.27 | 7.44 |
| Metabolism and nutrition disorders | Decreased appetite | 50 | 5.68 | 4.29 | 7.51 |
|  | Hypervolaemia | 4 | 6.92 | 2.59 | 18.46 |
|  | Hypoalbuminaemia | 22 | 86.50 | 56.77 | 131.82 |
|  | Malnutrition | 4 | 11.54 | 4.33 | 30.79 |
| Musculoskeletal and connective tissue disorders | Myalgia | 21 | 3.62 | 2.36 | 5.56 |
| Neoplasms benign, malignant and unspecified (incl cysts and polyps) | Acute myeloid leukaemia* | 3 | 5.41 | 1.74 | 16.80 |
|  | Myelodysplastic syndrome* | 6 | 12.93 | 5.80 | 28.83 |
|  | Plasma cell myeloma* | 22 | 12.45 | 8.18 | 18.95 |
|  | Plasma cell myeloma recurrent* | 6 | 39.62 | 17.76 | 88.38 |
| Nervous system disorders | Altered state of consciousness | 19 | 24.26 | 15.44 | 38.12 |
|  | Aphasia | 4 | 3.85 | 1.44 | 10.27 |
|  | Balance disorder | 18 | 5.86 | 3.68 | 9.31 |
|  | Coma | 6 | 3.62 | 1.62 | 8.06 |
|  | Encephalopathy | 6 | 6.83 | 3.06 | 15.21 |
|  | Immune effector cell-associated neurotoxicity syndrome | 100 | 568.28 | 463.27 | 697.09 |
|  | Neurological symptom | 3 | 15.87 | 5.11 | 49.28 |
|  | Neurotoxicity | 55 | 75.10 | 57.43 | 98.19 |
|  | Parkinsonism | 7 | 21.94 | 10.44 | 46.11 |
|  | Seizure | 18 | 3.28 | 2.06 | 5.21 |
|  | Somnolence | 32 | 4.50 | 3.18 | 6.38 |
|  | Tremor | 25 | 4.51 | 3.04 | 6.69 |
|  | Unresponsive to stimuli | 3 | 3.73 | 1.20 | 11.58 |
| Psychiatric disorders | Agitation | 9 | 3.93 | 2.04 | 7.56 |
|  | Hallucination, visual | 3 | 4.11 | 1.32 | 12.75 |
|  | Mental status changes | 4 | 5.09 | 1.91 | 13.57 |
| Renal and urinary disorders | Acute kidney injury | 33 | 4.02 | 2.85 | 5.67 |
|  | Incontinence | 5 | 13.86 | 5.76 | 33.35 |
| Respiratory, thoracic and mediastinal disorders | Hypoxia | 11 | 8.63 | 4.77 | 15.61 |
|  | Laryngeal oedema | 5 | 26.57 | 11.04 | 63.96 |
|  | Tachypnoea | 5 | 9.98 | 4.15 | 24.01 |
| Surgical and medical procedures | Lymphocyte adoptive therapy | 11 | 871.65 | 472.71 | 1607.28 |
| Vascular disorders | Hypotension | 39 | 5.41 | 3.94 | 7.42 |
|  | Shock | 3 | 4.19 | 1.35 | 13.01 |

Notes: * indicates that comparison with the drug instruction revealed "unexpected signals" for ide-cel.

**Supplementary Table 5** Signal strength of cilta-cel associated reports at the preferred terms level

| SOC | Preferred terms (PT) | Cases  (n) | ROR | 95% CI | |
| --- | --- | --- | --- | --- | --- |
|  |  |  |  | Low bound | Up  bound |
| Blood and lymphatic system disorders | Cytopenia | 7 | 18.30 | 8.71 | 38.47 |
|  | Febrile neutropenia | 8 | 4.67 | 2.33 | 9.36 |
|  | Neutropenia | 13 | 3.39 | 1.96 | 5.85 |
|  | Thrombocytopenia | 11 | 4.08 | 2.25 | 7.38 |
| Eye disorders | Diplopia | 8 | 14.15 | 7.06 | 28.35 |
| Gastrointestinal disorders | Colitis | 6 | 6.06 | 2.72 | 13.51 |
|  | Large intestine perforation* | 3 | 18.57 | 5.98 | 57.69 |
| General disorders and administration site conditions | Adverse drug reaction | 11 | 4.39 | 2.43 | 7.95 |
|  | Adverse event | 25 | 11.85 | 7.98 | 17.59 |
|  | Disease progression | 19 | 6.52 | 4.15 | 10.26 |
|  | Multiple organ dysfunction syndrome | 4 | 4.11 | 1.54 | 10.97 |
|  | Pyrexia | 58 | 7.14 | 5.49 | 9.28 |
| Immune system disorders | Cytokine release syndrome | 158 | 353.52 | 299.59 | 417.17 |
|  | Haemophagocytic lymphohistiocytosis | 18 | 66.89 | 42.00 | 106.53 |
|  | Hypogammaglobulinaemia | 3 | 18.21 | 5.86 | 56.55 |
|  | Immunodeficiency | 3 | 6.77 | 2.18 | 21.00 |
| Infections and infestations | Bacterial infection | 3 | 6.64 | 2.14 | 20.60 |
|  | Clostridium difficile infection | 3 | 4.66 | 1.50 | 14.47 |
|  | Covid-19 pneumonia | 11 | 21.55 | 11.91 | 39.00 |
|  | Cytomegalovirus infection reactivation | 3 | 25.03 | 8.06 | 77.75 |
|  | Escherichia sepsis | 3 | 45.82 | 14.74 | 142.41 |
|  | Haematological infection | 3 | 76.12 | 24.47 | 236.81 |
|  | Haemophilus sepsis | 3 | 2877.52 | 854.21 | 9693.36 |
|  | Lower respiratory tract infection | 5 | 3.99 | 1.66 | 9.59 |
|  | Metapneumovirus pneumonia | 3 | 1251.10 | 388.70 | 4026.84 |
|  | Rhinovirus infection | 3 | 25.53 | 8.22 | 79.31 |
|  | Sepsis | 15 | 5.58 | 3.36 | 9.28 |
|  | Septic shock | 7 | 6.66 | 3.17 | 13.99 |
|  | Viral infection | 5 | 6.33 | 2.63 | 15.23 |
| Investigations | Alanine aminotransferase abnormal | 5 | 89.00 | 36.92 | 214.57 |
|  | Aspartate aminotransferase increased | 9 | 8.95 | 4.65 | 17.23 |
|  | Blood lactate dehydrogenase increased* | 5 | 16.27 | 6.76 | 39.16 |
|  | Blood pressure abnormal | 3 | 5.45 | 1.76 | 16.92 |
|  | Full blood count abnormal | 9 | 9.65 | 5.01 | 18.59 |
|  | Laboratory test abnormal | 6 | 6.60 | 2.96 | 14.71 |
|  | Platelet count decreased | 11 | 3.98 | 2.20 | 7.19 |
|  | Sars-cov-2 test positive | 7 | 11.30 | 5.38 | 23.74 |
|  | Serum ferritin increased | 3 | 24.98 | 8.04 | 77.58 |
|  | White blood cell count increased | 3 | 3.69 | 1.19 | 11.46 |
| Injury, poisoning and procedural complications | Infusion related reaction | 9 | 5.02 | 2.61 | 9.66 |
| Metabolism and nutrition disorders | Hypoalbuminaemia | 3 | 17.34 | 5.58 | 53.84 |
| Nervous system disorders | Ageusia* | 4 | 6.65 | 2.49 | 17.75 |
|  | Anosmia* | 3 | 10.65 | 3.43 | 33.05 |
|  | Aphasia | 3 | 4.30 | 1.39 | 13.35 |
|  | Bell's palsy | 18 | 397.03 | 248.32 | 634.80 |
|  | Chronic inflammatory demyelinating polyradiculoneuropathy | 3 | 78.51 | 25.24 | 244.26 |
|  | Cranial nerve paralysis | 5 | 950.87 | 386.86 | 2337.19 |
|  | Encephalopathy | 5 | 8.48 | 3.52 | 20.40 |
|  | Facial nerve disorder | 7 | 650.35 | 305.72 | 1383.48 |
|  | Facial paralysis | 21 | 67.92 | 44.13 | 104.55 |
|  | Facial paresis | 6 | 75.37 | 33.76 | 168.28 |
|  | Guillain-barre syndrome | 4 | 35.66 | 13.36 | 95.22 |
|  | Haemorrhage intracranial | 5 | 13.78 | 5.72 | 33.16 |
|  | Hypersomnia | 3 | 4.28 | 1.38 | 13.29 |
|  | Iiird nerve disorder | 4 | 1448.72 | 523.71 | 4007.56 |
|  | Immune effector cell-associated neurotoxicity syndrome | 45 | 366.85 | 272.02 | 494.74 |
|  | Nervous system disorder | 5 | 10.90 | 4.53 | 26.23 |
|  | Neurotoxicity | 34 | 68.86 | 48.99 | 96.78 |
|  | Parkinsonism | 22 | 104.22 | 68.35 | 158.92 |
|  | Peripheral motor neuropathy | 3 | 87.46 | 28.10 | 272.17 |
|  | Peripheral sensory neuropathy | 4 | 27.98 | 10.48 | 74.70 |
| Neoplasms benign, malignant and unspecified (incl cysts and polyps) | Acute myeloid leukaemia | 3 | 8.07 | 2.60 | 25.05 |
|  | Myelodysplastic syndrome | 8 | 25.76 | 12.85 | 51.62 |
|  | Plasma cell myeloma* | 16 | 13.49 | 8.24 | 22.08 |
| Psychiatric disorders | Mental status changes | 4 | 7.58 | 2.84 | 20.23 |
| Product issues | Physical product label issue* | 3 | 100.26 | 32.21 | 312.12 |
|  | Product container issue | 4 | 15.91 | 5.96 | 42.46 |
|  | Product label issue* | 11 | 58.69 | 32.41 | 106.30 |
|  | Product packaging issue | 6 | 23.03 | 10.33 | 51.37 |
| Respiratory, thoracic and mediastinal disorders | Respiratory failure | 8 | 4.91 | 2.45 | 9.83 |
| Surgical and medical procedures | Hospitalisation | 16 | 3.62 | 2.21 | 5.93 |
|  | Lymphocyte adoptive therapy | 11 | 1301.15 | 705.17 | 2400.84 |
|  | Transfusion | 6 | 21.43 | 9.61 | 47.79 |

Notes: * indicates that comparison with the drug instruction revealed "unexpected signals" for cilta-cel.

**Supplementary Table 6** Characteristics of death outcome reports with ide-cel and cilta-cel

| **Characteristics** | **Reports, N (%)** | |
| --- | --- | --- |
|  | Ide-cel | Cilta-cel |
| **Overall** | N=71 | N=80 |
| **Sex** |  |  |
| Female | 26(36.6) | 13(16.3) |
| Male | 37(52.1) | 40(50.0) |
| Unknown or missing | 8(11.3) | 27(33.8) |
| **Weight, kg** |  |  |
| ＜50 | 1(1.4) | 0 |
| 50-100 | 36(50.7) | 31(38.8) |
| ＞100 | 4(5.6) | 5(6.3) |
| Unknown or missing | 30(42.3) | 44(55.0) |
| **Age group, years** |  |  |
| 18-64 | 29(40.8) | 20(25.0) |
| 65-84 | 32(45.1) | 27(33.8) |
| Unknown or missing | 10(14.1) | 33(41.3) |
| **Reporter** |  |  |
| Healthcare professionals | 62(87.3) | 65(81.3) |
| Consumers | 6(8.5) | 10(12.5) |
| Unknown or missing | 3(4.2) | 5(6.3) |
| **Reporting country** |  |  |
| United States | 45(63.4) | 65(81.3) |
| Other country | 26(36.6) | 15(18.7) |
| Unknown or missing | 0 | 0 |

**Supplementary Table 7** Ide-cel- and cilta-cel-related AEs that occurring one year post-administration

| Drug | SOC | PT | Time  (Day) |
| --- | --- | --- | --- |
| Ide-cel | Neoplasms benign, malignant and unspecified (incl cysts and polyps) | Myelodysplastic syndrome | 2872 |
|  | Renal and urinary disorders | Haematuria | 2402 |
|  | Neoplasms benign, malignant and unspecified (incl cysts and polyps) | Bowen's disease | 1136 |
|  | Neoplasms benign, malignant and unspecified (incl cysts and polyps) | Squamous cell carcinoma of skin | 737 |
|  | Neoplasms benign, malignant and unspecified (incl cysts and polyps) | Metastatic squamous cell carcinoma | 527 |
|  | Infections and infestations | Pneumonia pseudomonal | 518 |
|  | Infections and infestations | Septic shock | 518 |
|  | Neoplasms benign, malignant and unspecified (incl cysts and polyps) | Myelodysplastic syndrome | 476 |
|  | Renal and urinary disorders | Haematuria | 460 |
|  | Neoplasms benign, malignant and unspecified (incl cysts and polyps) | Bladder cancer | 460 |
|  | Nervous system disorders | Headache | 378 |
|  | Cardiac disorders | Bradycardia | 378 |
|  | Neoplasms benign, malignant and unspecified (incl cysts and polyps) | Plasma cell myeloma | 374 |
| Cilta-cel | Neoplasms benign, malignant and unspecified (incl cysts and polyps) | Prostate cancer | 1070 |
|  | Neoplasms benign, malignant and unspecified (incl cysts and polyps) | Malignant melanoma | 877 |
|  | Neoplasms benign, malignant and unspecified (incl cysts and polyps) | Myelodysplastic syndrome | 869 |
|  | Infections and infestations | Pneumonia pseudomonal | 861 |
|  | Infections and infestations | Covid-19 pneumonia | 861 |
|  | Neoplasms benign, malignant and unspecified (incl cysts and polyps) | Basal cell carcinoma | 828 |
|  | Infections and infestations | Lower respiratory tract infection | 794 |
|  | Infections and infestations | Gastroenteritis | 770 |
|  | Infections and infestations | Escherichia sepsis | 714 |
|  | Infections and infestations | Sepsis | 714 |
|  | Nervous system disorders | Spinal cord compression | 714 |
|  | Infections and infestations | Neutropenic sepsis | 625 |
|  | General disorders and administration site conditions | Asthenia | 575 |
|  | General disorders and administration site conditions | Fatigue | 575 |
|  | Skin and subcutaneous tissue disorders | Alopecia | 575 |
|  | Infections and infestations | Gastrointestinal viral infection | 575 |
|  | Musculoskeletal and connective tissue disorders | Arthritis | 575 |
|  | Metabolism and nutrition disorders | Decreased appetite | 575 |
|  | Neoplasms benign, malignant and unspecified (incl cysts and polyps) | Plasma cell myeloma in remission | 575 |
|  | Investigations | Blood pressure decreased | 575 |
|  | Immune system disorders | Cytokine release syndrome | 575 |
|  | Neoplasms benign, malignant and unspecified (incl cysts and polyps) | Plasma cell myeloma refractory | 575 |
|  | Infections and infestations | Pneumonia influenzal | 466 |
|  | Infections and infestations | Metapneumovirus infection | 466 |
|  | Infections and infestations | Herpes zoster | 435 |
|  | Infections and infestations | Pneumonia | 424 |
|  | General disorders and administration site conditions | Pyrexia | 419 |
|  | Cardiac disorders | Acute coronary syndrome | 419 |
|  | Infections and infestations | Metapneumovirus pneumonia | 419 |
|  | Nervous system disorders | Transient ischaemic attack | 402 |

**
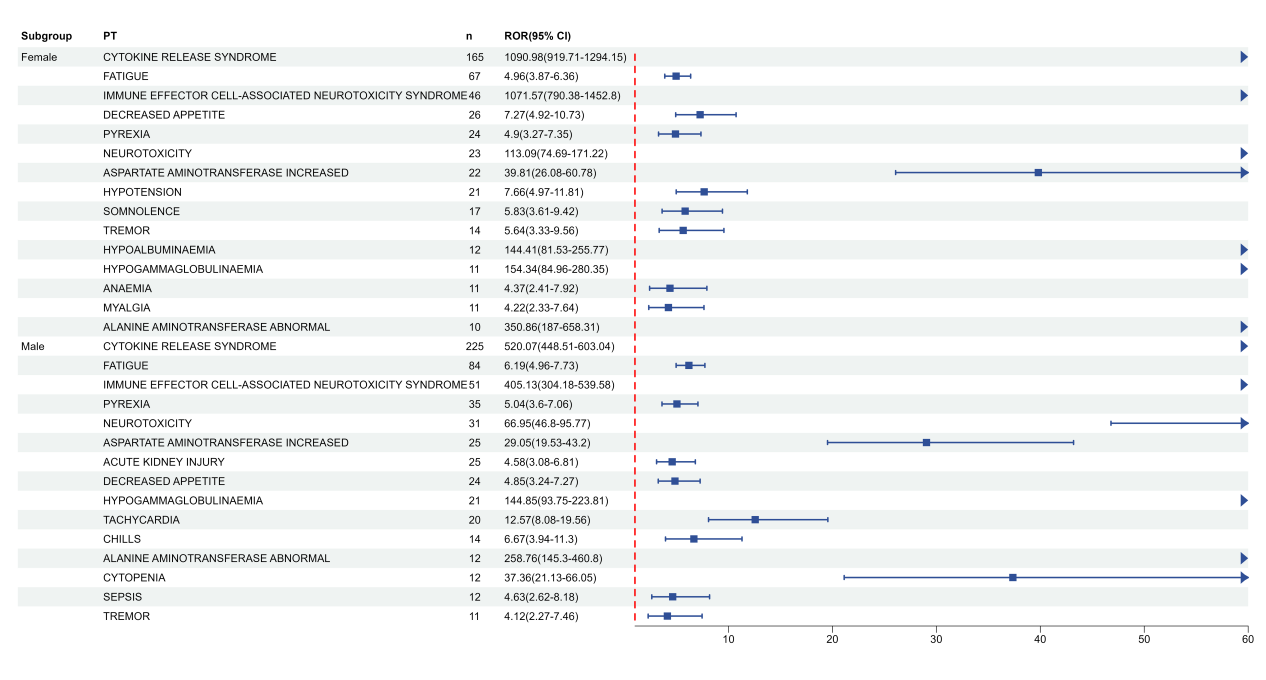
**

**Supplementary Figure 1.** Disproportionality analysis of sex subgroups in ide-cel


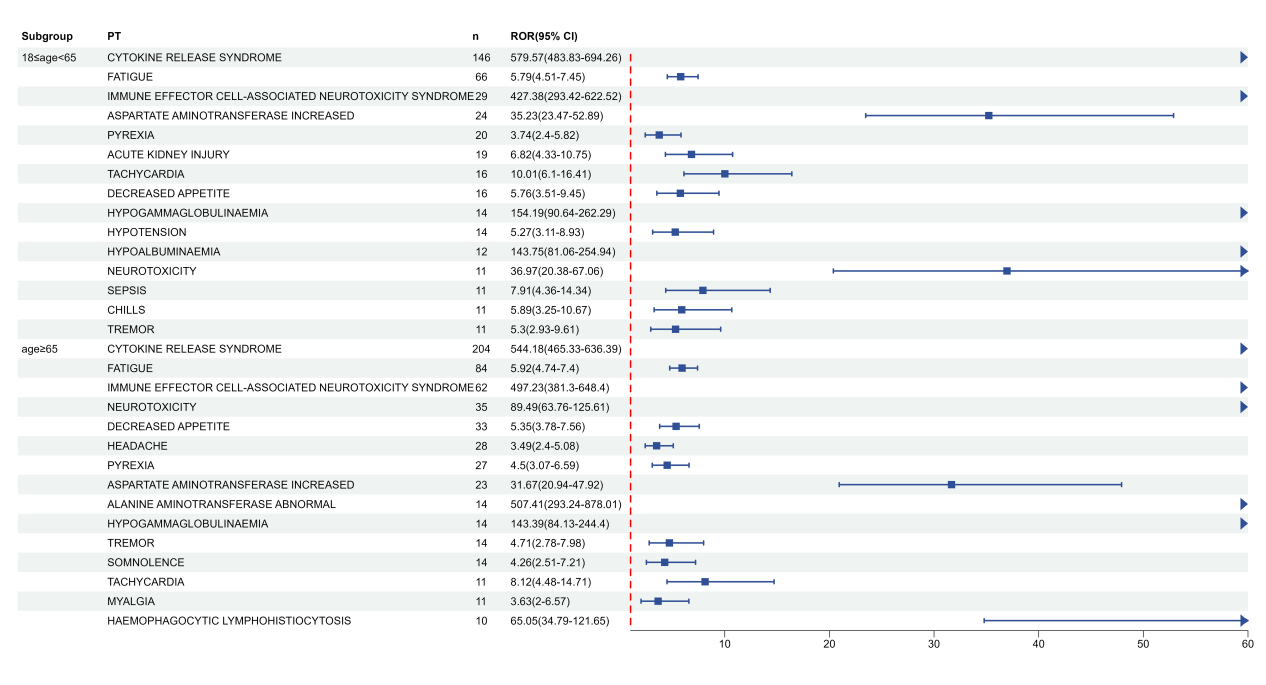


**Supplementary Figure 2.** Disproportionality analysis of age subgroups in ide-cel

**
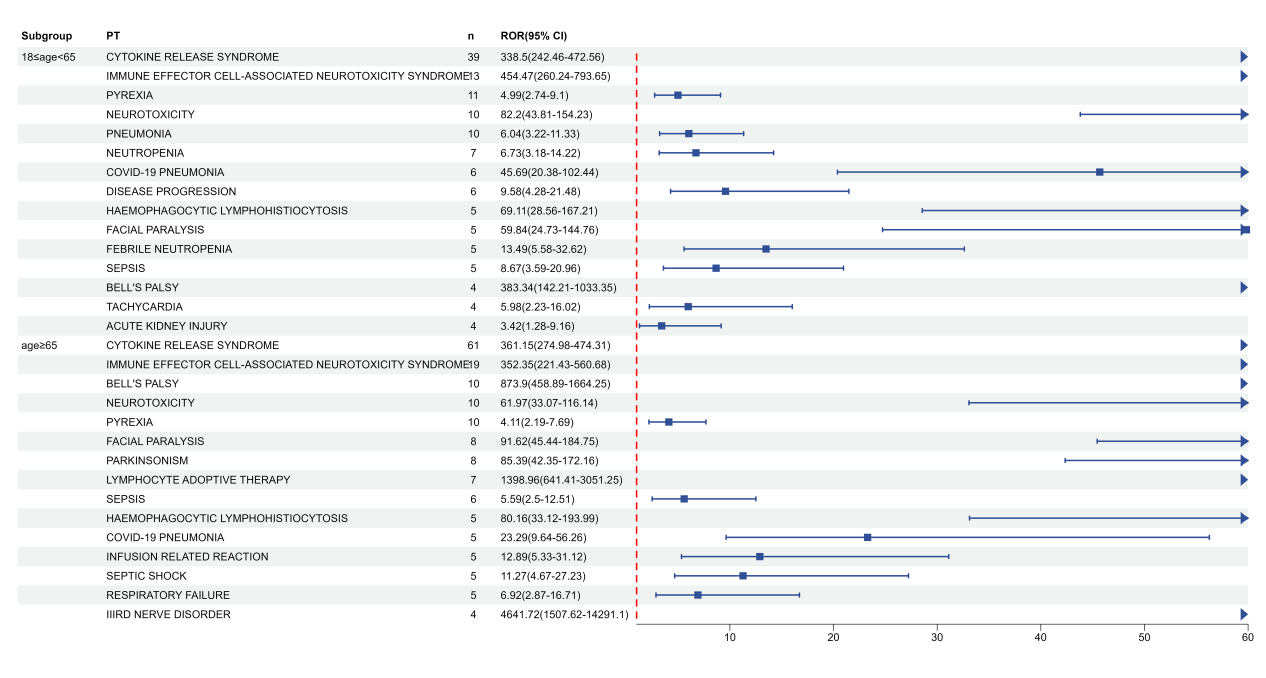
Supplementary Figure 3.** Disproportionality analysis of sex subgroups in cilta-cel


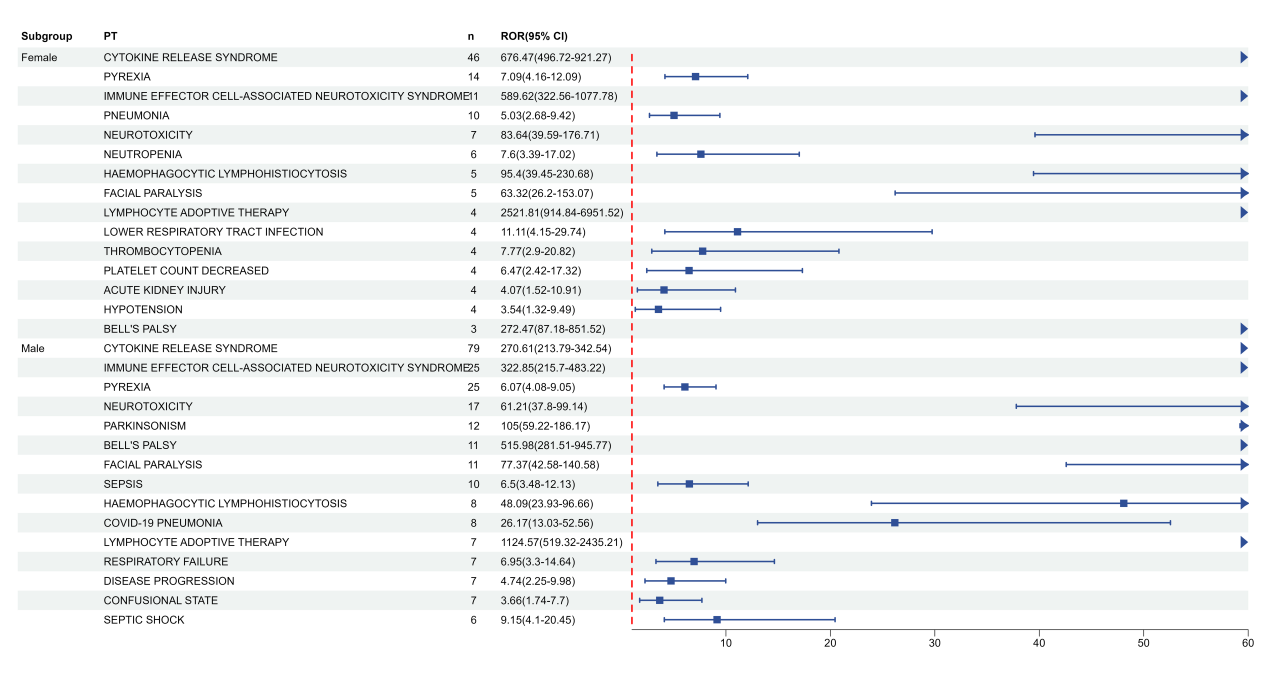


**Supplementary Figure 4.** Disproportionality analysis of age subgroups in cilta-cel
